# Duration of labor stages and pregnancy outcomes in vaginal birth after cesarean: a retrospective comparative analysis

**DOI:** 10.3389/fmed.2025.1643142

**Published:** 2025-11-06

**Authors:** Tianying Zhu, Dan Luo, Yan Li, Liling Xiong

**Affiliations:** Chengdu Women’s and Children’s Central Hospital, School of Medicine, University of Electronic Science and Technology of China, Chengdu, China

**Keywords:** labor duration, vaginal birth after cesarean, pregnancy outcomes, postpartumhemorrhage, epidural anesthesia

## Abstract

**Objective:**

This study aimed to characterize the labor duration distribution and evaluate maternal-neonatal outcomes of vaginal birth after cesarean (VBAC) under epidural anesthesia.

**Method:**

In this retrospective comparative study, we analyzed 156 term singleton VBAC cases with epidural anesthesia at Chengdu Women’s and Children’s Central Hospital (January 2021–December 2024), matched 1:1 by age to nulliparous controls. Comparative analyses of baseline characteristics, pregnancy complications, delivery modes, pregnancy outcomes, and labor durations were performed using independent *t*-tests, Mann–Whitney U tests, and *χ*^2^/Fisher’s exact tests. VBAC cases were stratified by prior labor attempt (*n* = 25 with vs. *n* = 131 without) to assess its impact on labor progression, with Spearman tests evaluating inter-stage correlations.

**Results:**

Demonstrated that VBAC cases had significantly higher median 24 h postpartum blood loss (330 vs. 250 mL), postpartum hemorrhage rates (43% vs. 20%), and neonatal NICU admission rates (10% vs. 2%) compared to nulliparous controls (all *p* < 0.05). The 95th percentile durations for VBAC were 730 min (first stage) and 81 min (second stage), both significantly shorter than controls (*p* < 0.05), while third-stage durations were comparable (95th percentile, 11 vs. 10 min, *p* > 0.05). Prior labor attempt did not influence VBAC labor progression (*p* > 0.05). Positive correlations existed between first and second stages in both groups (*r* = 0.297, *p* = 0.002).

**Conclusion:**

These results suggest that VBAC under epidural anesthesia may progress faster through first and second stages of labor than nulliparous deliveries but carries higher risks of adverse outcomes. Clinical management should integrate multifactorial assessment, warranting further investigation into labor patterns and outcome relationships.

## 1 Introduction

The global cesarean delivery rate has risen steadily since the 1990s, becoming a major public health concern (1). China exemplifies this trend, with its national cesarean rate increasing from 41.6% in 2016 (2) to 44.5% in 2020 (3)—a surge attributable to evolving fertility policies and the growing proportion of advanced maternal age pregnancies. In this context of high cesarean rates coupled with the need for fertility preservation, optimizing delivery management for women with prior cesareans has emerged as a critical priority in perinatal medicine worldwide.

The concept of trial of labor after cesarean (TOLAC) was introduced in the United States during the 1970s to challenge the prevailing dogma of “once a cesarean, always a cesarean” (4). Promoted by the American College of Obstetricians and Gynecologists (ACOG) and the National Institutes of Health (NIH), TOLAC rates peaked at 28.9% in 1996. However, subsequent reports of uterine rupture cases led to stricter guidelines in 2010 for vaginal birth after cesarean (VBAC) (5). Current data indicates that approximately 21% of eligible women attempt TOLAC in the U.S., with a success rate of 85% (6, 7), underscoring the importance of meticulous labor management in achieving safe VBAC.

The progression of labor duration standards has provided an important reference for TOLAC management. In 1955, Friedman established the classic “S”-shaped labor curve based on data from 500 term deliveries, which guided clinical practice for half a century. His study defined the active phase as starting at 3 cm cervical dilation, with a recommended maximum second stage of 2 h for nulliparous women (8). However, in 2010, Zhang et al.’s large-scale study of 62,415 parturients challenged this standard. Their findings showed that labor progression was slower before 4–6 cm dilation, suggesting the active phase may actually begin at 6 cm. Additionally, they reported that the 95th percentile for the second stage was 3.6 h with epidural analgesia and 2.8 h without it for nulliparous women (9). These insights led to the 2012 revision of labor guidelines by Maternal-Fetal Medicine (SMFM) (10). Nevertheless, debates persist regarding whether prolonged labor increases risks such as postpartum hemorrhage and infection (11, 12). Moreover, there is limited evidence on whether these updated labor standards apply to TOLAC populations. Notably, current TOLAC labor studies primarily involve Western populations. Given China’s higher cesarean delivery rate, there is an urgent need to establish labor management strategies tailored to its unique demographic characteristics.

To address these critical evidence gaps, we conducted a systematic evaluation of VBAC cases at our tertiary referral center, analyzing both maternal-neonatal safety outcomes and labor progression patterns to inform evidence-based TOLAC management in high-cesarean-rate settings such as China.

## 2 Materials and methods

### 2.1 Study design and participants

**Figure 1:**
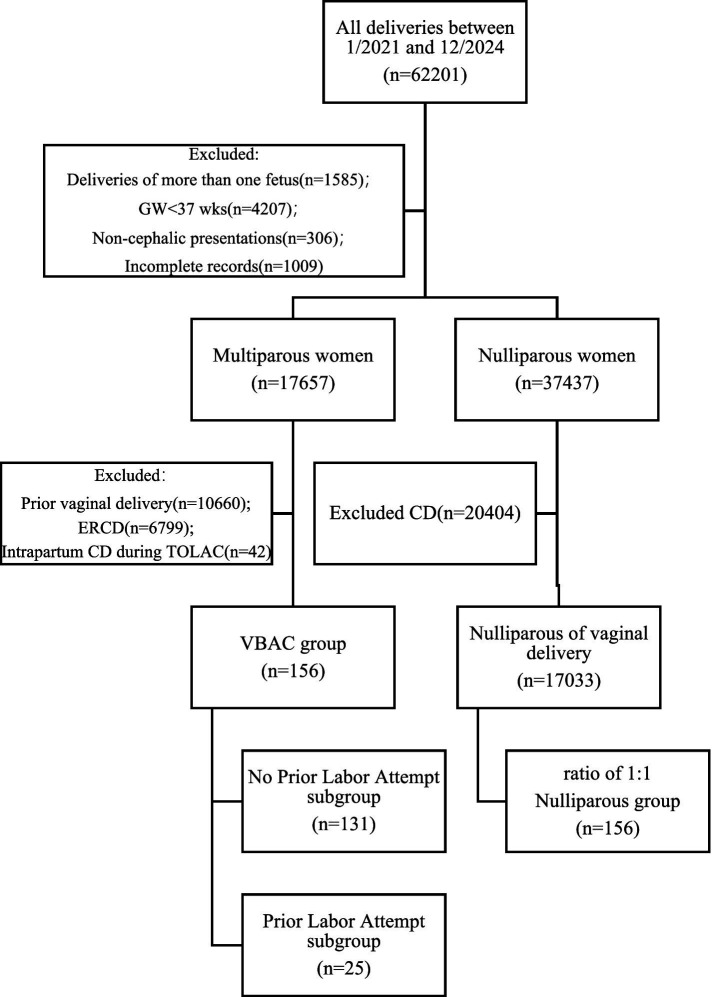
Study population enrollment flowchart. During the study period, a total of over 60,000 deliveries occurred. After screening based on the inclusion and exclusion criteria, a total of 156 VBAC cases were included as the study group, and 156 term nulliparous women delivering during the same period were selected as controls.

This retrospective comparative study evaluated outcomes between two predefined groups: women with successful vaginal birth after cesarean (VBAC) and age-matched nulliparous women with spontaneous vaginal delivery at Chengdu Women’s and Children’s Central Hospital from January 1, 2021, to December 31, 2024. During the study period, a total of over 60,000 deliveries occurred. Inclusion Criteria: singleton pregnancy, term delivery (gestational age ≥37 weeks), cephalic presentation, no prior vaginal delivery and complete clinical records. Exclusion criteria included multiple gestation, preterm birth (<37 weeks), non-cephalic presentation, incomplete records, prior vaginal delivery, elective repeat cesarean (ERCD), or intrapartum cesarean during TOLAC. A total of 156 VBAC cases were included as the study group. To enable meaningful comparison, 156 term nulliparous women who achieved spontaneous vaginal delivery and met the same inclusion criteria of the study group during the same period were selected as controls (1:1 matching by maternal age ±2 years) (Figure 1).

### 2.2 Management

#### 2.2.1 Labor induction

Gestational age was confirmed by first-trimester ultrasound (8–12 weeks). Induction methods were determined by senior obstetricians based on Bishop score: for cases with Bishop score <6, cervical ripening was performed using a double-balloon catheter (DBC), followed by oxytocin induction if required; for cases with Bishop score ≥6, artificial rupture of membranes (AROM) and/or low-dose oxytocin infusion was administered.

#### 2.2.2 Intrapartum management

All TOLAC patients received continuous fetal monitoring, intravenous access, and bedside electrocardiogram monitoring. Clear liquid intake was permitted, with urinary catheterization and routine blood tests (including blood type and screen) performed. The labor unit maintained 24/7 availability of attending obstetricians, anesthesiologists, and senior midwives, with immediate readiness for emergent cesarean delivery. For cases with inadequate contractions (defined as <3 contractions per 10 min, each lasting <45 s), labor augmentation was achieved through low-dose oxytocin infusion and/or AROM. Operative vaginal delivery (forceps-assisted) was utilized when indicated. Uterine integrity was assessed via pre-and postpartum ultrasound evaluation of the lower uterine segment.

### 2.3 Data collection

Maternal variables included: age (continuous, years); gestational age at delivery (weeks, confirmed by first-trimester ultrasound); pre-pregnancy BMI (kg/m^2^, categorized as <24 vs. ≥24 based on Chinese obesity criteria); gestational weight gain (kg, continuous); pregnancy complications (binary: present/absent for hypertensive disorders of pregnancy (HDP), diabetes, anemia, thyroid dysfunction); labor induction method (categorized as: double-balloon catheter, artificial rupture of membranes [AROM], oxytocin, or none); delivery mode (spontaneous vaginal vs. forceps-assisted); postpartum hemorrhage (PPH, defined as blood loss ≥500 mL within 24 h); and uterine rupture (clinical + ultrasound-confirmed). Neonatal variables included birth weight (grams), 5-min Apgar score (<7 vs. ≥7), and NICU admission (yes/no). Labor durations were recorded in minutes for first (onset of regular contractions to full dilation), second (full dilation to delivery), and third stages (delivery to placental expulsion).

All participants received standardized intrapartum care per institutional TOLAC protocol, including continuous fetal monitoring, readiness for emergent cesarean delivery, and postpartum uterine integrity assessment via ultrasound.

### 2.4 Group stratification

The primary analysis compared the VBAC group (*n* = 156) with age-matched nulliparous controls (*n* = 156). A secondary subgroup analysis was performed within the VBAC cohort, stratifying cases by indication for prior cesarean delivery: prior failed labor (*n* = 25) versus elective cesarean (*n* = 131). Labor duration distributions and inter-stage correlations were evaluated across all groups.

### 2.5 Statistical analysis

Statistical analyses were conducted using SPSS version 22.0. Normally distributed continuous variables were expressed as mean ± standard deviation and compared using independent t-tests. Non-normally distributed data were presented as median (interquartile range) and analyzed with Mann–Whitney U tests. Categorical variables were reported as frequencies (percentages) and compared using *χ*^2^ or Fisher’s exact tests, as appropriate. Correlations between labor stages were assessed using Pearson’s test. A two-tailed *p*-value <0.05 was considered statistically significant.

## 3 Results

During the study period, there were 7,099 pregnancies with a history of cesarean section, among which 198 cases (2.79%) underwent TOLAC. A total of 156 cases achieved VBAC, yielding a success rate of 78.79%. Uterine rupture occurred in 1 case (0.5%).

### 3.1 General characteristics and outcomes

Compared to the nulliparous group, the VBAC group demonstrated significantly higher gestational age at delivery and higher proportion of deliveries ≥40 weeks, but lower gestational weight gain (*p* < 0.05 for all comparisons). Regarding pregnancy complications, the VBAC group had higher incidence of diabetes but lower incidence of anemia compared to nulliparous women (*p* < 0.05 for both comparisons). In terms of labor induction methods, the VBAC group showed higher utilization rate of artificial rupture of membranes (*p* < 0.05) (Table 1).

**Table 1:** Comparative analysis of baseline characteristics, pregnancy complications, and labor induction methods between VBAC and nulliparous groups.

Variable	VBAC group	Nulliparous group	*t*/*χ*^2^	*p*
*N*	156	156		
Maternal age (mean±SD)	33.07 ± 3.15	32.61 ± 4.06	−0.951	0.343
≥35 years (%)	59 (37.8)	56 (35.9)	0.124	0.407
Gestational age at delivery (mean±SD)	38.83 ± 0.82	39.34 ± 0.84	4.595	<0.001
≥40 weeks (%)	14 (9.0)	50 (32.1)	25.476	<0.001
Weight gain (kg)	12.48 ± 4.10	14.33 ± 4.36	3.222	0.001
BMI ≥ 24 (pre-pregnancy; %)	11 (7.1)	14 (9.0)	0.391	0.393
PROM (%)	36 (23.1)	27 (17.3)	1.611	0.13
HDP (%)	36 (23.1)	20 (13.3)	4.856	0.019
Diabetes in pregnancy (%)	7 (4.5)	11 (7.1)	0.943	0.234
Anemia during pregnancy (%)	13 (8.3)	29 (18.6)	7.043	0.006
Thyroid dysfunction during pregnancy (%)	23 (14.7)	19 (12.2)	0.44	0.31
Induction of labor (%)	68 (43.6)	56 (35.9)	1.927	0.102
CRB (%)	30 (19.2)	19 (12.2)	2.929	0.06
AROM (%)	44 (28.2)	20 (12.8)	11.323	0.001
Oxytocin (%)	53 (34.0)	41 (26.3)	2.192	0.087

BMI, Body Mass Index; PROM, Prelabor Rupture of Membranes; HDP, Hypertensive Disorders of Pregnancy; CRB, Cervical Ripening Balloon (i.e., double-balloon catheter); AROM, Artificial Rupture of Membranes.

For maternal outcomes, the VBAC group exhibited significantly greater postpartum blood loss and higher risk of postpartum hemorrhage. Neonatal outcomes revealed higher NICU admission rates in the VBAC group compared to nulliparous controls (*p* < 0.05 for all comparisons) (Table 2).

**Table 2:** Comparison of delivery modes and neonatal outcomes between VBAC and nulliparous groups.

Variable	VBAC group	Nulliparous group	*χ*^2^ / *Z*	*p*
*N*	156	156		
Forceps-Assisted Birth (%)	11 (7.1)	4 (2.6)	3.385	0.056
Spontaneous vaginal delivery (%)	145 (92.9)	152 (97.4)	3.385	0.056
24 h postpartum blood loss (mL, median [IQR])	330 (250 ~ 400)	250 (195 ~ 335)	−5.275	<0.001
PPH (%)	43 (27.6)	20 (12.8)	10.521	0.001
Uterine rupture (%)	1 (0.5)	0	1.003	1
Birth weight (g, median [IQR])	3,170 (2,990 ~ 3,420)	3,280 (3,080 ~ 3,420)	−1.338	0.181
5-min Apgar scores<7 (%)	1 (0.6)	1 (0.6)	0	1
Admission in NICU (%)	10 (6.4)	2 (1.3)	5.547	0.035

PPH, Postpartum Hemorrhage; NICU, Neonatal Intensive Care Unit.

### 3.2 Labor duration distribution and correlation analysis

Both first and second stages of labor were significantly shorter in the VBAC group compared to nulliparous women with epidural anesthesia (*p* < 0.05). The median (Interquartile Range, IQR) first stage duration was 290 (218–450) minutes in VBAC group (95th percentile, 730 min) versus 365 (247.5–547.5) minutes (95th percentile, 720 min) in nulliparous group. For the second stage, VBAC group showed median duration of 27 (16–43.5) minutes (95th percentile, 81 min) compared to 34 (17–65) minutes (95th percentile, 127.5 min) in nulliparous group. Third stage durations were comparable between groups (Table 3).

**Table 3:** Comparison of labor stage durations between VBAC and nulliparous groups.

Length of the labor	VBAC group	Nulliparous group	*Z*	*p*
*N*	156	156		
First stage (min)				
Median (IQR)	290 (218 ~ 450)	365 (247.5 ~ 547.5)	−2.779	0.005
95th Percentile	730	720		
Second stage (min)				
Median (IQR)	27 (16 ~ 43.5)	34 (17 ~ 65)	−2.024	0.043
95th Percentile	81	127.5		
Third stage (min)				
Median (IQR)	6 (4 ~ 7)	5 (5 ~ 7)	−0.898	0.369
95th Percentile	11	10		

IQR, Interquartile Range.

To evaluate potential confounding from prior labor experience, we stratified VBAC cases into subgroups with (*n* = 25) and without (*n* = 131) history of labor attempt. No significant differences in labor durations were observed between these subgroups. The 95th percentiles for first, second and third stages were 757 vs. 742 min, 75.2 vs. 83.2 min, and 10.8 vs. 11 min in subgroups with and without prior labor experience, respectively (Table 4).

**Table 4:** Comparison of labor stage durations between VBAC subgroups with and without prior labor attempt.

Labor stage	Prior labor attempt	No prior labor attempt	*Z*	*p*
*N*	25	131		
First stage (min)				
Median (IQR)	300 (245 ~ 465)	275 (197.25 ~ 446.25)	−0.88	0.379
95th Percentile	757	742		
Second stage (min)				
Median (IQR)	27 (16 ~ 46)	26.5 (15.75 ~ 43.25)	−0.131	0.896
95th Percentile	75.2	83.2		
Third stage (min)				
Median (IQR)	5 (4 ~ 7)	6 (5 ~ 8)	−1.931	0.369
95th Percentile	10.8	11		

IQR, Interquartile Range.

### 3.3 Correlation analysis of labor stages

In VBAC group, significant correlation was observed between first and second stages (*r* = 0.297, *p* = 0.002), while no significant correlations existed between second and third stages (*r* = 0.097, *p* = 0.315) or first and third stages (*r* = 0.002, *p* = 0.672). Similarly, nulliparous group showed correlation between first and second stages (*r* = 0.232, *p* = 0.015) but not between other stage combinations (first-third: *r* = 0.027, *p* = 0.777; second-third: *r* = −0.099, *p* = 0.305).

## 4 Discussion

### 4.1 Antenatal management of VBAC

Current evidence indicates that the success rate of TOLAC in achieving VBAC ranges between 60 and 80% (13), with appropriate antenatal management being a crucial determinant of successful outcomes. Multiple studies have demonstrated that macrosomia (fetal weight ≥4,000 g) and maternal obesity (BMI ≥ 30 kg/m^2^) significantly reduce TOLAC success rates while increasing the risk of uterine rupture by 1.5- to 2-fold (14, 15). These associations may be closely related to gestational weight gain (GWG). The 1990 Institute of Medicine (IOM) report first systematically established the strong relationship between GWG and neonatal birth weight, showing that excessive GWG increases risks of macrosomia and dystocia, whereas overly restricted GWG may elevate risks of preterm birth and low birth weight (16). Consequently, researchers worldwide have explored various GWG management models, though no universal standard has been established due to variations in ethnicity, dietary culture, and socioeconomic status. The widely accepted approach involves individualized GWG targets based on pre-pregnancy BMI stratification (e.g., the 2009 IOM guidelines), although its applicability to TOLAC populations remains controversial. Our study found that the average GWG in the VBAC group (12.48 kg) was significantly lower than in nulliparous women (14.33 kg, *p* < 0.05), contrasting with Li et al.’s findings (3), which reported no difference (both groups ~14 kg). Although our TOLAC success rate (76.22%) surpassed that of Li’s study (68%), regional dietary differences preclude definitive conclusions regarding stricter GWG control for TOLAC. Future multicenter studies are needed to investigate GWG thresholds affecting VBAC success across BMI categories and to develop ethnicity-specific GWG management models.

### 4.2 Maternal and neonatal outcomes of VBAC

TOLAC offers women with prior cesarean sections the option of vaginal delivery, avoiding the risks associated with repeat cesarean sections and reducing the likelihood of placenta accreta spectrum disorders in subsequent pregnancies. However, our study revealed that the VBAC group had significantly higher 24-h postpartum blood loss (*p* < 0.001), postpartum hemorrhage rates (27.6% vs. 12.8%), and neonatal NICU admission rates (6.4% vs. 1.3%) compared to nulliparous women (*p* < 0.05), consistent with findings from Li and Zhou et al. (3, 17). Li suggested that with increasing parity, uterine muscle tone decreases, thereby elevating the risk of postpartum hemorrhage (PPH) in the VBAC group (3). Supporting this, a retrospective cohort study by Chen et al. (18) in China demonstrated that women with a uterine scar are at significantly increased risk of PPH. This heightened susceptibility is likely attributable to structural and functional uterine impairment resulting from the prior cesarean section, including compromised myometrial contractility and defective placental site involution during the index pregnancy (18). Furthermore, our study observed higher rates of forceps-assisted delivery and AROM in the VBAC group compared to the control group. These interventions may contribute to increased birth trauma and infection risk, potentially explaining the elevated NICU admission rates among neonates in the VBAC cohort. One case of uterine rupture occurred during the study (incidence: 0.5%), presenting as sudden fetal heart rate deceleration during labor. The diagnosis was confirmed via ultrasound following forceps-assisted delivery, and immediate surgical repair was performed, resulting in favorable maternal and neonatal outcomes. This underscores two key ACOG recommendations (4): (1) TOLAC must be conducted in centers capable of emergency cesarean delivery, and (2) standardized emergency protocols are essential. Although our center maintained uterine rupture rates at the lower end of guideline-reported ranges (0.5–0.9%) through strict GWG control and prenatal ultrasound assessment of the lower uterine segment, clinicians must remain vigilant for the classic triad of “acute abdominal pain, abnormal fetal monitoring, and hematuria.” We propose risk-stratified management for TOLAC candidates: low-risk women (e.g., prior vaginal delivery, spontaneous labor onset) may undergo intensified labor monitoring, while high-risk women (e.g., induction, labor arrest) should provide informed consent for potential emergency surgery to balance VBAC benefits and perinatal safety.

### 4.3 Analysis of labor duration

Considerable controversy persists regarding labor duration in VBAC, with limited large-scale data available. Hila Shalev-Ram et al.’s Israeli study of 422 VBAC cases (19) reported that the partogram was similar to those of nulliparous vaginal deliveries without epidural analgesia, a finding echoed by Graseck et al.’s U.S. study of 140 VBAC cases (20). In contrast, Li et al.’s analysis of 359 VBAC cases from Hubei, China (3), demonstrated significantly shorter median first-stage duration in VBAC (390 min) versus nulliparous women (450 min), a result corroborated by our study even after excluding women with prior labor attempts. This divergence likely reflects population differences in labor progression, as Graseck’s cohort included parous women with vaginal delivery experience. Our findings, achieved through strict standardization of oxytocin administration and exclusion of women with prior labor attempts, align with Li’s data and imply unique VBAC labor characteristics in Asian women. Notably, our VBAC group exhibited shorter first-stage durations than previous studies, potentially attributable to stricter GWG management (resulting in lower birth weights) and higher rates of artificial rupture of membranes (28.2% vs. 12.8%). The latter accelerates labor by increasing prostaglandin synthesis through lysosomal release of phospholipase A2 from amniotic cells. This also reflects heightened clinical attention to TOLAC cases, indicating that intervention intensity significantly impacts labor duration. However, neither this study nor Li’s research included labor curve analysis. Future studies should incorporate the new labor standards to better characterize cervical dilation patterns in TOLAC.

Regarding the second stage, international evidence remains inconsistent. A multicenter randomized controlled trial of 790 TOLAC cases from Ireland, Italy, and Germany associated shorter labor durations with higher TOLAC success rates (21), a conclusion supported by Mark P et al. (22) and Gabriel Levi et al. (23), who further identified significantly increased risks of adverse outcomes when the second stage exceeded 3 h. Conversely, G. Gitas et al.’s German study of 1,546 VBAC cases (24) found no significant differences in second-stage duration or outcomes before and after implementing the new labor guidelines, questioning their utility in TOLAC management. Discrepancies also exist in comparisons between VBAC and nulliparous women: Hila Shalev-Ram et al. (19) reported significantly shorter second stages in VBAC without epidural analgesia (19 vs. 47 min, *p* = 0.023), but this difference disappeared with epidural use (81 vs. 111 min, *p* = 0.34), possibly due to analgesia-induced reductions in PGE2 levels. In contrast, Gabriel Levin et al.’s study of 1,310 VBAC cases (25) demonstrated consistently shorter second stages in VBAC regardless of analgesia (overall median: 88 vs. 103 min; with epidural: 105 vs. 118 min; without epidural: 28 vs. 44 min; all *p* < 0.01). Our findings similarly confirmed shorter second stages in VBAC. Hila Shalev-Ram (19) suggested that even when the previous delivery was by cesarean section, the functional capacity of the birth canal may have changed, resulting in a shorter second stage compared to nulliparous women. On the other hand, due to the risk of uterine rupture in TOLAC, healthcare providers often provide more intensive labor monitoring, which may lead to a shortened second stage. These differences indicate that when developing TOLAC labor management strategies, it is necessary to comprehensively consider influencing factors such as analgesia methods and the intensity of labor interventions.

In addition to investigating the durations of all three labor stages, this study also analyzed the correlations between them. The results demonstrated a significant correlation between the duration of the first stage and that of the second stage, further validating the finding that both the first and second stages were significantly shorter in the VBAC group compared to the nulliparous group. This observation suggests that in clinical practice, prolonged first-stage labor should alert clinicians to the possibility of subsequent second-stage prolongation, warranting enhanced maternal-fetal monitoring.

### 4.4 Limitations

Our study has several limitations. Firstly, our study is a retrospective design and may have potential biases (e.g., selection bias, unmeasured confounders), in our future research, we hope to conduct a prospective study to further verify our research results. Secondly, the study’s findings may lack generalizability due to its single-center setting and modest sample size. To address this limitation, future research could adopt a multi-center approach and include a more diverse population to enhance the external validity of the results. Additionally, conducting a meta-analysis of similar studies could provide a broader perspective and improve the generalizability of the findings.

## 5 Conclusion

TOLAC labor management presents unique challenges and requires healthcare institutions to have comprehensive emergency resources and multidisciplinary collaboration capabilities. This study confirmed that VBAC parturients exhibit significantly different labor characteristics compared to nulliparous women, manifesting as markedly shorter durations of both first and second stages. However, labor management should not focus solely on temporal parameters. Instead, a multidimensional evaluation system should be established, where clinicians conduct comprehensive and dynamic assessments of maternal-fetal status. Clinical decision-making must also integrate multiple factors, including fetal monitoring results, labor progression trends, and maternal general condition. Currently, there remains significant disagreement in the global evidence base regarding TOLAC labor management, with particularly scarce high-quality research data focusing on Asian populations. Therefore, large-scale, multicenter prospective studies are urgently needed to establish an evidence-based TOLAC labor evaluation system that can provide more precise guidance for clinical practice.

## Data Availability

The original contributions presented in the study are included in the article/supplementary material, further inquiries can be directed to the corresponding author.
